# MRSA carriage among healthcare workers in non-outbreak settings in Europe and the United States: a systematic review

**DOI:** 10.1186/1471-2334-14-363

**Published:** 2014-07-03

**Authors:** Madeleine Dulon, Claudia Peters, Anja Schablon, Albert Nienhaus

**Affiliations:** 1 Department of Occupational Health Research, Institution for Statutory Accident Insurance and Prevention in the Health and Welfare Services, Pappelallee 33/35/37, 22089 Hamburg, Germany; 2 Institute for Health Services Research in Dermatology and Nursing, University Medical Centre Hamburg-Eppendorf, Martinistr. 52, 20246 Hamburg, Germany

**Keywords:** MRSA, Colonisation, Healthcare worker

## Abstract

**Abstarct:**

**Background**

A recent review estimated prevalence of methicillin-resistant *Staphylococcus aureus* (MRSA) in healthcare workers (HCWs) to be 4.6%. However, MRSA carriage in HCWs in non-outbreak settings is thought to be higher than in an outbreak situation, due to increased hygiene awareness in outbreaks, but valid data are missing. The goals of this paper are to summarise the prevalence of MRSA carriage amongst HCWs in non-outbreak situations and to identify occupational groups in healthcare services associated with a higher risk of MRSA colonisation.

**Methods**

A systematic search for literature was conducted in the MEDLINE and EMBASE databases. The methodological quality of the studies was assessed using seven criteria. Pooled prevalence rates were calculated. Pooled effect estimates were identified in a meta-analysis.

**Results**

31 studies were included in this review. The pooled MRSA colonisation rate was 1.8% (95% confidence interval [CI], 1.34%-2.50%). The rate increased to 4.4% (95% CI, 3.98%-4.88%) when one study from the Netherlands was excluded. The pooled MRSA rate was highest in nursing staff (6.9%). Nursing staff had an odds ratio of 1.72 (95% CI, 1.07-2.77) when compared with medical staff and an odds ratio of 2.58 (95%, 1.83-3.66) when compared with other healthcare staff. Seven studies were assessed as being of high quality. The pooled MRSA prevalence in high quality studies was 1.1% or 5.4% if the one large study from the Netherlands is not considered. The pooled prevalence in studies of moderate quality was 4.0%.

**Conclusions**

MRSA prevalence among HCWs in non-outbreak settings was no higher than carriage rates estimated for outbreaks. Our estimate is in the lower half of the range of the published MRSA rates in the endemic setting. Our findings demonstrate that nursing staff have an increased risk for MRSA colonisation. In order to confirm this finding, more studies are needed, including healthcare professionals with varying degrees of exposure to MRSA. In order to reduce misclassification bias, standardisation of HCWs screening is warranted.

## Background

Methicillin-resistant *Staphylococcus aureus* (MRSA) is the most commonly identified antimicrobial-resistant pathogen in hospitals in many parts of the world [1]. In Europe, the proportion of methicillin resistance in strains of *Staphylococcus aureus* (*S. aureus*) isolates in infected patients varied in 2011 from less than 0.5% to more than 50%, with a pooled mean rate of around 17% [1]. In the United States, the proportion of methicillin resistance in *S. aureus* strains approached almost 60% in 2003, with an average rate of resistance over the period 1998–2002 of around 50% [2]. In several European countries, a reduction in the proportion of bloodstream infections caused by MRSA has been observed, which may reflect the success of infection control measures in the clinical setting [3]. Nevertheless, the burden of healthcare-associated MRSA colonisation seems to extend beyond the clinical setting to long-term care facilities and outpatient care [4].

The anterior nares are the main reservoir of MRSA, although other body sites are frequently colonised, such as the hands, skin, axillae, and intestinal tract [5,6]. Colonised individuals are generally asymptomatic and three types of MRSA carrier status can be distinguished: non-carriers, persistent carriers, who are chronically colonised with the same strain, and intermittent carriers, who are colonised with varying strains for short time periods [6]. A special form of short-term carriage is transient carriage, which is identified during or after a work shift and in most cases lost before the next shift [7]. Nasal carriage of *S. aureus* has been associated with an increased risk of infection for the colonised individual [8], and a similar increased risk is expected for intestinal carriage [5]. However, it is unclear whether the risk of infection is higher for the colonised individual when carriage is persistent [9,10]. Approximately 5% of colonised HCWs develop clinical infections [6] and symptomatic MRSA infections among HCWs have been described in several case reports [11,12].

Healthcare workers (HCWs) are likely to be important in the transmission of MRSA, but more frequently act as vectors, rather than being the main sources of MRSA transmission [6,13,14]. The most important mode of MRSA transmission is through contamination of the hand [15]. An alternative mechanism of transmission is airborne dispersal of staphylococci in association with an upper respiratory tract infection [16]. Colonised HCW are most often transiently colonised, but they may become persistent carriers if they have chronic dermatitis or sinusitis, and this may lead to prolonged MRSA transmission [17,18].

Whilst routine screening of all potential inpatients at risk is receiving increasing political support, the procedures of screening and decolonisation for colonised HCWs remain controversial [6,13]. Although in regions with low MRSA prevalence, such as the Netherlands, screening after each contact with MRSA-positive patients is recommended [19], the guidelines of several European countries and North American health associations are more reluctant and only advocate staff screening in selected situations, such as epidemiological outbreaks [17,20-22]. Decolonisation of nasal colonised HCWs with mupirocin is recommended by most guidelines, but critical questions have arisen about the systematic use of this antibacterial agent [23]. Other issues related to the management of colonised HCW have been raised in the literature, including the questions of the optimum timing of HCW screening and whether and for how long colonised HCWs should be excluded from work [6,13]. Work restrictions for HCWs colonised with MRSA differ geographically, ranging from being allowed to work without restrictions other than compulsory hand hygiene, to being removed from clinical duties or being forced to take leave of absence [6].

In German speaking countries, active post-exposure screening of HCWs is not routine [21,24,25]. In the case of persistent carriers, further employment of the employee is not advised where there is patient contact. The German Code of Social Law (SGB VII, Art 9, §3) does allow the recognition of an infection with MRSA as an occupational disease, however colonisation with MRSA without signs of infection cannot be recognised and compensated for as an occupational disease. HCWs with MRSA colonisation might therefore suffer from job restrictions without being covered by the social accident insurance.

The prevalence of MRSA colonisation among HCWs was assessed in two reviews to be around 5%, on the basis of 127 papers published between 1980 and March 2006 [6] and a further 18 papers published between April 2006 and March 2010 [13]. Both reviews included worldwide MRSA data from endemic situations and outbreaks. Aside from outbreaks, it is assumed that MRSA rates will be higher when HCWs comply poorly with hand hygiene and contact precautions, as they are not fully aware of the threat of the bacteria load [6]. However, only a few data are available on the prevalence of MRSA carriers among HCWs in non-outbreak settings. The goals of the present review were to document the prevalence of MRSA carriage amongst HCWs in non-outbreak settings in European countries and the United States and to identify occupational groups and specialties in the healthcare services associated with a higher risk of MRSA exposure.

## Methods

### Search strategy and screening

The systematic review was conducted according to the PRISMA statement [26]. We searched MEDLINE and EMBASE for articles published between January 2000 and December 2013. Search terms and Medical subject Headings (MeSH) for Methicillin-resistant *Staphylococcus aureus* and health personnel were combined with two searches relating to prevalence and screening as follows:

•Methicillin-Resistant *Staphylococcus aureus* OR MRSA

AND

•Health personnel OR healthcare worker$ OR healthcare professional$ OR staff OR employees

AND

•Prevalence OR cross-sectional study OR carrier state OR carriage

AND

•Screening OR screen OR surveillance

The results of the search were limited to studies in human and to articles written in English, German, Spanish or Italian. The titles and abstracts identified by the search were screened by MD and at least by one of the other authors and relevant papers were selected for the review. Full-text evaluation was conducted by MD and in case of uncertainty, discussion took place with the other authors. Articles were included if they reported prevalence rates of MRSA in personnel working in healthcare settings in European countries or the United States. We restricted our search to studies carried out in European countries and the United States, as the situation in healthcare settings in these countries can be compared directly. Prospective (cohort) studies were included if baseline data were presented for the whole study population. Articles were excluded if they were related to outbreaks, to patients or residents only, were not performed in Europe or in the United States, or were designed as an incidence study. Bibliographies of included studies were also reviewed to retrieve any further references. Results were not limited to peer-reviewed publications, e.g., Letters to the Editor and abstracts of conference publications were also included.

A data extraction form was developed to collect information on the following points: 1) study design (country, study period, sample size, study population, and healthcare setting); 2) swabbing methods (anatomic sampling sites and screening strategy); 3) results (number and percentage of subjects colonised with MRSA and percentage of MRSA carriage related to occupational groups or specialty); and 4) study quality.

As we conducted a review of existing literature no approval of an ethics committee was required.

### Assessment of study quality

The quality of included papers was independently assessed by two authors (CP and AS), using a tool composed of seven criteria taken from a checklist aimed at evaluating the quality of prevalence surveys [27] and the STROBE statement [28]. The criteria were expressed as questions (Table 1). The answers were graded as *Yes* (with a score of one point) if the question was satisfactorily answered, otherwise with a score of zero points if information was missing (not documented) or unclear. Study quality was assessed as high (>4 points) or moderate (≤4 points).

**Table 1:** Checklist for the quality assessment of MRSA prevalence surveys in healthcare workers

**Number**	Criteria*	**Content**
1	Specification of the target population	Are study subjects and the setting described?
2	Adequate sample size**	Is the sample size adequate?
3	Adequate response rate	Is the response rate adequate - at least 60%?
4	Information on non-responders	Are the non-responders described?
5	Valid and repeatable disease definition	Are standard measures (microbiological and molecular typing methods) used for detection of MRSA?
6	Bias	Are efforts described to address potential sources of bias and/or have potential sources of bias been discussed?
7	Interpretation of the results	Were confidence intervals or standard errors presented for the estimates of prevalence?

*Criteria adopted from [27,28].

**Calculation of adequate sample size was based on the following assumptions: prevalence rate of MRSA in healthcare workers = 5%, error rate ±2.5 at the 95% confidence level, resulting in a sample size of 284; we considered a final sample size (after subtracting the non-responders) to be of 250.

### Statistical analysis

Studies were grouped according to the quality level (high and moderate), study area (United States and Europe), and occupational group (nursing staff, medical staff and other healthcare staff). The pooled prevalence of MRSA colonisation was calculated by dividing the number of MRSA-colonised subjects by the total number of subjects for whom culture results were reported. Carriage rates were described by 95% confidence intervals of proportions. Studies which reported stratified numerator and denominator data for occupational groups were included in the meta-analysis. The proportion of subjects in two occupational groups (nursing and medical staff) was compared with the group of other healthcare staff and with each other. These data were used to calculate odds ratios (OR) as effect estimates and 95% confidence intervals (95% CI). For the purpose of meta-analysis, a combined effect estimate was calculated using the Mantel-Haenszel method for dichotomous outcomes. Odds ratios were stratified for study area and study quality. χ^2^ analysis was used to compare proportions and to determine heterogeneity among studies. As the χ^2^ test has a low sensitivity for detecting heterogeneity in the situation of a meta-analysis when studies have a small sample size, *P* value of < .1 was considered significant for the presence of statistical heterogeneity [29]. In case of homogeneity, we used a variance approach with a fixed effect model and in case of heterogeneity we used the random effect model [29]. Publication bias due to study size was assessed by a funnel plot [30]. The analysis was carried out using Review Manager (RevMan 5.1).

## Results

### Studies identified and assessment of study quality

**Figure 1:**
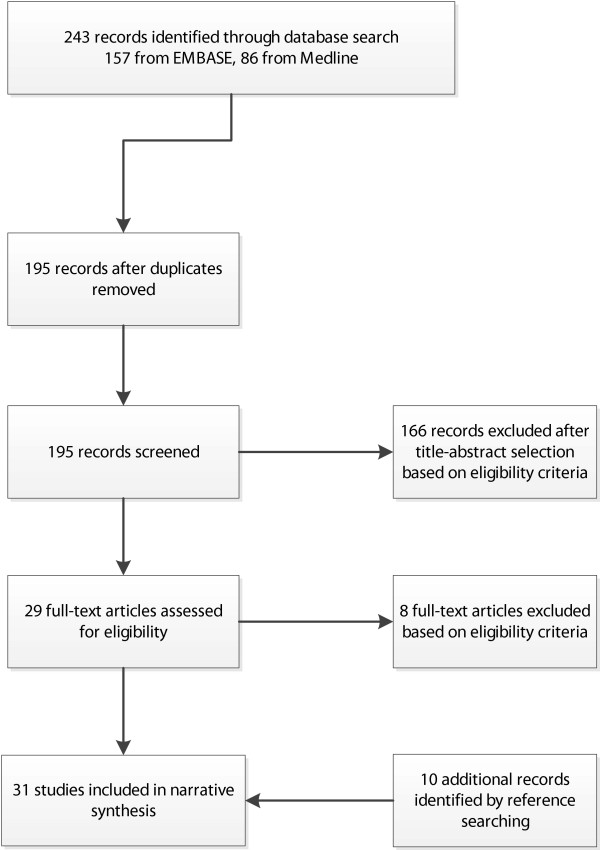
**Study-selection process for this review.** PRISMA flowchart.

The database search identified 195 unique papers (Figure 1). A total of 166 papers were excluded, because data of MRSA rates were collected outside Europe or the United States (n = 32), were collected during outbreaks (n = 19), belonged to patients or residents only (n = 25), or because the topics of these papers were reviews or guidelines or focused on other subjects (incidence studies, cost calculations, MRSA associated with livestock or communities) (n = 90). Twenty nine papers were eligible for full-text evaluation, of which 21 were included. An additional ten papers were identified via reference screening. Thus, a total of 31 papers were included in the review (Table 2).

**Table 2:** **Studies reporting methicillin-resistant *Staphylococcus aureus *** **(MRSA) colonisation in healthcare workers (HCWs) in non-outbreak situations in Europe and The United States**

**Reference**	**Study year (duration of the screening period)**	**Country**	**Study population**		**Swabbing methods**		**Results**		**Quality scoring**^**b**^
			**Sample size**	**Profession, Healthcare setting**^**a**^	**Anatomic sampling sites**	**Screening strategy**	**No. (%) of subjects with MRSA**	**Related to occupation or specialty - % of subjects with MRSA**	
**Quality level: high**									
Baldwin 2009 [31]	2005-2006 (9 months)	Northern Ireland	563	Nurses, care and domestic assistants, kitchen and clerical staff; nursing home (n = 45)	Nose	On one day in each nursing home	43 (7.5)	Nurses 8.7	1:Y; 2:Y;
								Care assistants 10.2	3:Y; 4:N;
								Others 3.5	5:Y; 6:Y;
									7:Y
Elie-Turenne 2010 [32]	2008 (4 weeks)	US, New York	256	Nurses/therapists, physicians, clerical staff, paramedics; hospital (emergency department (ED), ICU, pre-hospital emergency medical service (EMS)	Nose	Swabs taken between 5:00–11:00 to gain participation of staff who worked during night, day & mid-day shifts	17 (6.6)	Nurses/ther. 10.5	1:Y; 2:Y;
								Physicians 3.8	3:N; 4:N;
								Clerical 3.6	5:Y; 6:Y;
								Paramedics 1.9	7:Y
								ED 9.6	
								ICU 5.1	
								EMS 1.9	
Eveillard 2004 [33]	1998-1999 (7 months)	France	965	Nursing, medical, laboratory, radiology, engineering and administrative staff; hospital (ICU, medical ward, LTCF-E)	Nose	ND	60 (6.2)	Nursing 9.6	1:Y; 2:Y;
								Medical 6.3	3:Y; 4:N;
								Lab./radio. 2.7	5:Y; 6:Y;
								Engineering 2.0	7:Y
								Admin. 0.8	
								LTCF-E 36.0	
								Medical ward 12.5	
								ICU 3.0	
Jager 2010 [34]	2008 (4 months)	The Netherlands	266	Employees; medical microbiology laboratory (n = 5)	Nose, throat	Self-swabbing	1 (0.4)		1:Y; 2:Y;
									3:Y; 4:N;
									5:Y; 6:Y;
									7:N
Johnston 2007 [35]	2004-2005 (6 months)	US, Baltimore	200	Nurses and physicians; hospital (medical ICU and inpatient HIV infection unit)	Nose	Once a month	4 (2.0)^c^		1:Y; 2:N;
							8 (4.0)^d^		3:Y; 4:N;
									5:Y; 6:Y;
									7:Y
Suffoletto 2008 [36]	2006 (3 months)	US, Pittsburgh	255	Nurses, nursing assistants, radiology and respiratory technicians, physicians, clerical staff; hospital (n = 5 (emergency departments)	Nose	Swabs taken at the beginning of the shift	11 (4.3)	Nursing/medical	1:Y; 2:Y;
								technicians 6.9	3:N; 4:N;
								Physicians 0.0	5:Y; 6:Y;
								Clerical 0.0	7:Y
Vos 2009 [37]	2000-2004 (4 years)^c^	The Netherlands	13.195	HCWs involved in the care of MRSA positive patients; hospital (medical centre)	Nose, throat, skin lesions	Routine personnel screening	31 (0.2)^d^		1:Y; 2:Y;
									3:Y; 4:N;
									5:Y; 6:Y;
									7:N
**Quality level: moderate**									
Amorim 2009 [38]	2003-2005 (26 months)	Portugal	126	HCWs (nurses, nurse aids, physician, administrative and technical staff); hospital (vascular surgery, endocrinology ward)	Nose	3 screening sessions per year	6 (4.8)^c^	Nurses 8.9	1:Y; 2:N;
								Nurse aids 4.4	3:Y; 4:N;
								Physicians 2.5	5:Y; 6:Y;
								Admin/techn 0.0	7:N
Becker 2013 [39]	ND	Germany	13	Nursing staff; LTCF-E (n = 2)	Nose	ND	1 (7.7)		1:Y; 2:N;
									3:N; 4:N;
									5:Y; 6:N;
									7:N
Berthelot 2004 [40]	2000 (1 month)	France	333	Fellows, nursing and medical students; control group of HCWs; hospital	Nose	ND	14 (2.5)	Students 2.4	1:Y; 2:Y;
								Control group 2.6	3:N; 4:N;
									5:N; 6:N;
									7:Y
Bisaga 2008 [41]	2006 (5 months)	US, Chicago	105	Nurses, physicians, technicians; hospital (emergency department)	Nose	Swabs taken during the shift at times convenient to the subjects	16 (15.0)	Nurses 17.0	1:Y; 2:N;
								Physicians 8.0	3:N; 4:N;
								Technicians 18.0	5:Y; 6:Y;
									7:Y
Boisseau 2012 [42]	2010 (1 day)	France	152	Physicians, paramedical HCWs; attendees of a medical conference	Nose	ND	4 (2.4)		1:Y; 2:N;
									3:N; 4:N;
									5:Y; 6:Y;
									7:N
Brady 2009 [43]	2008 (1 day)	United Kingdom	260	Surgical and non-surgical physicians (phys.); attendees of a medical conference (n = 2)	Nose	ND	6 (2.0)	Surgical phys. 4.8	1:Y; 2:Y;
								Non-surgical phys. 0.6	3:N; 4:N;
									5:N; 6:Y;
									7:N
Edmundson 2011 [44]	2005-2008 (4 years)	Ireland	566	Nurses, doctors, radio-graphers, occupational therapists, porters, physiotherapists; hospital (orthopaedic unit)	Nose	Twice a year as far as possible	27 (4.8)^c^		1:Y; 2:Y;
									3:N; 4:N;
									5:N; 6:Y;
									7:N
Garcia Lozano 2012 [45]	2011 (1 month)	Spain	62	HCWs involved in treat-ment of MRSA-positive patients; hospital; (ICU in an institute of oncology)	Nose, pharynx	ND	1 (1.6)		1:Y; 2:N;
									3:N; 4:N;
									5:Y; 6:N;
									7:N
Gruber 2013 [46]	2006-2007 (6 months)	Germany	64	Staff members; nursing home (n = 8), geriatric clinic (n = 2)	Nose, throat	ND	2 (3.1)	Nursing home 4.0	1:Y; 2:N;
								Geriatric clinic 0.0	3:N; 4:N;
									5:Y; 6:N;
									7:N
Heudorf 2002 [47]	2000-2001 (6 months)	Germany	158	Staff members; nursing home and geriatric rehabilitation unit	Nose, throat	ND	1 (0.6)		1:Y; 2:N;
									3:N; 4:N;
									5:N; 6:N;
									7:N
Ibarra 2008 [48]	2006-2007 (16 months)	US, South Texas	257	Nurses, physicians, other health care personnel; paediatric hospital	Nose	ND	30 (12.0)	Nurses 12.0	1:Y; 2:Y;
								Physicians 13.0	3:N; 4:N;
								Other HCWs 11.0	5:Y; 6:Y;
									7:N
Kaminski 2007 [49]	2001-2002 (4 months)	Germany	324	Nurses and physicians involved in the treatment of MRSA-positive patients; hospital (trauma unit)	Nose, throat	ND	17 (5.3)		1:Y; 2:Y;
									3:N; 4:N;
									5:Y; 6:N;
									7:N
Kampf 2003 [50]	1995-1996 (12 months)	Germany	447	Nursing and medical staff); hospital (ICU and general ward)	Nose, oro-pharynx	Episodal staff screening	3 (0.7)	Nursing 0.8	1:Y; 2:Y;
								Medical 0.0	3:N; 4:N;
								General ward 0.7	5:Y; 6:Y;
								ICU 0.6	7:N
March 2010 [51]	2008 (1 month)	Italy	69	Nurses, physicians and other staff; LTCF-E (n = 5)	Nose, oro-pharynx, inguinum	ND	10 (14.5)		1:Y; 2:N;
									3:Y; 4:N;
									5:Y; 6:N;
									7:N
Monaco 2009 [52]	2005 (2 days)	Italy	51	Staff members; nursing home	Nose	ND	3 (5.8)		1:Y; 2:N;
									3:N; 4:N;
									5:Y; 6:N;
									7:N
Neuhaus 2002 [53]	2000-2001 (12 months)	Germany	193	Nursing staff; nursing home (n = 61)	Nose, throat	ND	1 (0.5)		1:Y; 2:N;
									3:N; 4:N;
									5:Y; 6:N;
									7:Y
Nulens 2005 [54]	2003 (1 day)	United Kingdom	335	Physicians and non-physicians; attendees of a medical congress	Nose	Swabs taken by HCWs them-selves during the congress	1 (0.3)	Physicians 0.5	1:Y; 2:N;
								Non-physicians 0.0	3:N; 4:N;
									5:Y; 6:N;
									7:N
Orsi 2008 [55]	ND	Italy	200	Nurses and physicians; hospital (general ward, ICU, cardio- and neurological surgery unit)	Nose	Swabs taken at the beginning of the shift	3 (1.5)	Nurses 1.6	1:Y; 2:N;
								Physicians 1.3	3:N; 4:N;
									5:N; 6:Y;
									7:Y
Prior 2011 [56]	2003 [2003–2010]	Ireland	172^c^ [2.048]^d^	Nurses, physicians, other staff; hospital (orthopaedic surgery unit)	Nose	Routine pre-employment screening	3 (1.7)^c^ [47 (2.3)]^d^		1:Y; 2:Y;
									3:N; 4:N;
									5:N; 6:Y;
									7:N
Reich-Schupke 2010 [57]	2008 (8 weeks)	Germany	83	Employees; hospital (dermatological ward)	Nose	Swabs taken before starting work	4 (4.8)		1:Y; 2:N;
									3:N; 4:N;
									5:Y; 6:Y;
									7:N
Sassmannshausen 2011 [58]	2010 (ND)	Germany	726	HCWs (nurses, physicians, other staff); hospitals (n = 9)	Nose, pharynx	Swabs taken after a non-working weekend	33 (4.5) 23 (3.2%)^e^		1:Y; 2:Y;
									3:N; 4:N;
									5:N; 6:N;
									7:N
Scarnato 2003 [59]	2000 (12 months)	France	193	Nursing staff, physicians, physical therapists, office staff; geriatric wards (acute medical care and long-term care units)	Nose	Once a month	4 (2.1)^c^		1:Y; 2:N;
									3:N; 4:Y;
									5:Y; 6:Y;
									7:N
Schwarzkopf 2010 [60]	ND (12 months)	US, New York	135	Surgeons (resident and attending surgeons); hospital (orthopaedic surgery)	Nose	ND	2 (1.5)	Attending surgeons 2.7	1:Y; 2:N;
								Resident surgeons 0.0	3:Y; 4:Y;
									5:N; 6:N;
									7:N
Stahl 2011 [61]	ND	France	565	Physicians (phys.); attendees of medical conferences in France	Nose	ND	29 (5.1)^f^	Intensive care phys. 8.0	1:Y; 2:Y;
								Infect. disease phys. 2.4	3:N; 4:N;
								Hygiene phys. 1.6	5:Y; 6: N;
									7:N

^a^*Abbreviations*: *ICU* intensive care unit, *LTCF-E* long-term care facility for the elderly, *ND* not documented.

^b^Details for criteria see Table 1.

^c^Data related to the baseline screening.

^d^Data related to the total study period (with repeated screening surveys).

^e^Positive on two consecutive swabs.

^f^Number and percentage calculated by MD.

After consideration of the seven quality criteria, the quality level was assigned as “high” in seven studies [31-37] and as “moderate” in 24 studies [38-61]. Six of the 31 studies included were based in the United States and 25 in Europe, conducted in eight different countries (France, Germany, Ireland, Italy, Portugal, Spain, the Netherlands and the United Kingdom). Sample size ranged from 13 to 13,195, with a median sample size per study of 200 HCWs (interquartile range, 126–335). A variety of professions were included as participants (e.g. nurses, care assistants, physicians, paramedics, domestic staff, cleaning staff, kitchen staff and administrative staff). Study participants worked in around 14 different specialties. Around 60% of the studies (n = 18) were conducted in acute care hospitals, eight studies in long-term care facilities for the elderly, and one study in medical microbiology laboratories. Four studies were conducted in a non-clinical surrounding such as conferences [42,43,54,61]. All studies performed screening by nasal swabbing; some studies used additional sampling sites. Swabs were collected by research assistants except for two studies, in which swabs were taken by the HCWs themselves [34,54]. The screening strategy with regard to the timing of the swabbing was described by five studies [32,36,41,55,57]. Two studies presented data collected by routine screening [37,56].

A total of 21,289 subjects were included in the review. MRSA colonisation was identified in 388 of the HCWs swabbed, corresponding to a pooled prevalence of 1.8% (95% CI, 1.34%-2.50%). The carriage rate within the individual studies ranged from 0.2% to 15%. When the large study from the Netherlands [37] was excluded from the analysis (because more than 60% of subjects included in the review were supplied by this study), the pooled MRSA prevalence among the remaining 8,094 HCWs increased to 4.4% (95% CI, 3.98%-4.88%). The pooled MRSA prevalence among HCWs in Europe was 1.5% (or 4.0% when the study from the Netherlands was excluded) and was significantly lower than the value estimated for HCWs in the United States (*p*< 0.001), with a pooled prevalence of 6.6% (95% CI, 5.35%-8.17%). The pooled MSRA prevalence among HCWs screened in a non-clinical surrounding was 3.1% (95% CI, 2.24%-4.13%).

It was not possible to pool MRSA data for different specialties, as only a few papers focused on the same specialty. For specialities covered by at least three papers, the ranges were as follows: 0.5% to 36.0 for long-term care facilities for the elderly [31,33,39,46,47,51-53], 1.6% to 5.1% for intensive care units [32-34,45], and 4.3% to 15.0% for emergency departments [32,36,41].

The pooled MRSA prevalence among HCWs from the high quality studies was 1.1% (95% CI, 0.66%-1.74%) and was significantly lower than among HCWs from methodologically moderate studies (*p* < 0.001), with a pooled prevalence of 4.0% (95% CI, 3.47%-4.50%). When the study from the Netherlands was excluded, the pooled MRSA prevalence among HCWs from high quality studies increased to 5.4% (95% CI, 4.61%-6.39%).

**Figure 2:**
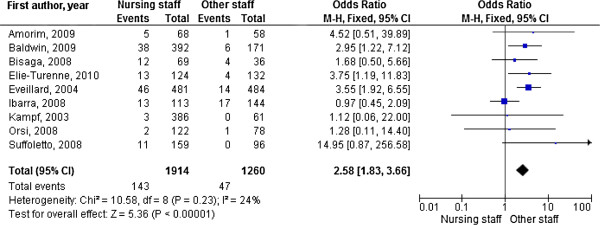
**Forest plot showing the risk of MRSA colonisation for healthcare workers.** Block with line = Odds ratio with 95% CI. For convenience reasons only the first author of the study is given.

The meta-analysis of all studies reporting MRSA prevalence rates for nursing staff (n = 8) showed that the risk of MRSA colonisation was 2.58 times higher than for other healthcare staff; the χ^2^ test showed no evidence of heterogeneity (Figure 2). Visual examination of the funnel plot to assess publication bias revealed no systematic relation between study size and the magnitude of the estimator (funnel plot not shown). When all high quality studies were pooled, the risk for nursing staff was even more pronounced than for other staff (OR 3.66). Nursing staff had an odds ratio of 1.72 when compared with medical staff (Table 3). Stratified analysis by study area showed that in Europe risk of MRSA colonisation was three times higher for nursing staff than for other healthcare staff (OR 3.19), whereas studies conducted in the United States provided no evidence of greater risk (Table 3).

**Table 3:** Pooled estimates of MRSA colonisation among healthcare workers: stratified analysis

**Stratified analysis [sets of studies]**	**Number of studies**	**Pooled estimation**		**Heterogeneity**	
		**OR**	**95% CI**	**Χ**^**2**^	**p value**
Nursing staff versus medical staff [32,33,36,38,41,48,50,55]	8	1.72	1.07-2.77	4.60	0.71
Nursing staff versus other healthcare staff					
- high-quality studies [31-33,36]	4	3.66	2.33-5.77	1.18	0.76
- study area Europe [31,33,38,50,55]	5	3.19	1.98-5.13	1.26	0.87
- study area US [32,36,41,48]	4	2.07	0.83-5.15	6.4	0.09

## Discussion

This review incorporated 31 studies on MRSA prevalence rates among HCWs in Europe and the United States. Only studies conducted in a non-outbreak setting were included. The pooled prevalence of MRSA colonisation among HCWs was estimated as 1.8%. When the study of Vos et al. [37] reporting data of routine screening from the Netherlands was excluded, pooled prevalence increased to 4.3%. This estimate is slightly lower than the result of a previously conducted review, which estimated the average MRSA carriage rate among HCWs as 4.6% [6]. Bearing in mind that estimates of Albrich and Harbarth [6] were based on worldwide data and in a variety of endemic and outbreak settings, the similarity between the results is surprising. The authors themselves pointed out that the studies on endemic settings and outbreaks were quite heterogenous [6], so that direct comparison does not appear appropriate. We believe that the methods used in non-outbreak studies included in our review permit a more valid estimation of MRSA prevalence, than studies performed during outbreaks which usually focus on the efficacy of the measures taken. Our estimate should be compared with published values in the endemic setting. It is towards the lower end of the range of 2% to 15% given in [13] and about half the value of 8.1% estimated by [6].

Our review has shown that MRSA carriage in HCWs varies widely over a range between under 1% and 15%. Carriage rates among HCWs are much higher than among community members without known risk factors (around 0.2% [62]). MRSA carriage was higher among HCWs in the United States than in Europe but carriage was not as high as could have been expected, if it is considered that the proportion of methicillin-resistance of *S. aureus* isolates in infected patients is three times higher in the USA than in Europe (60% vs. 20%) [1,2]. Within Europe, there is a north to south gradient, with less than 1% invasive MRSA infections in northern Europe and more than 25% in southern and south-eastern Europe [1]. Our data cannot be used to confirm this gradient for HCWs, as there are few studies from countries with very low or very high prevalence. In Italy, the MRSA prevalence in patients is between 25% and 50% [1], and MRSA carriage rates in HCWs varied between 1.5% and 14.5% (n = 3 studies).

Although study participants were in a variety of occupations, occupation-related carriage rates combined with absolute figures were reported in fewer than 10 studies and only for nurses and physicians. We found significantly elevated risks for nursing staff. According to our data, the risk for nursing staff of being colonised with MRSA was almost two-fold higher than for medical staff and three-fold higher than for other healthcare staff. To our knowledge, this comparison of pooled MRSA prevalence data between nursing and medical staff has not been conducted in other studies. Our finding is concordant with previous studies [32,38,41] and can be explained by the more frequent and close contact of nurses with patients than for other healthcare professionals. Other studies have reported poor associations between profession and MRSA carriage rate [33,48].

With regard to high risk units, the mean proportions of MRSA are apparently higher in staff working in emergency departments, though no significant trend was observed. It is unclear from our data whether non-clinical settings such as nursing homes or neurological rehabilitation units can be regarded as high risk units with regard to MRSA exposure for staff. But in Germany, a large proportion of claims to the compensation board for MRSA infections are made by nursing staff from nursing homes for the elderly [12].

The methodological quality of the studies was quite inconsistent with respect to sample size, response rate and efforts to address potential sources of bias. Only seven studies were assessed as being high-quality studies. Pooled MRSA carriage rate was higher among HCWs from high quality studies than for moderate-quality studies. There is no apparent explanation for this observation.

Levels of staff MRSA carriage in published studies are generally difficult to interpret. The most important reasons for misclassification are sampling site, and the frequency and timing of HCW screening [6,13]. Only the anterior nares were used as sampling sites in most studies, this could lead to underestimation of the true prevalence of MRSA carriage. The reliability of the result could also have been affected if the nasal samples were taken by different staff members, rather than just by one trained study assistant. The timing of the screening test will have an important influence on the results obtained, as MRSA colonisation is often transient - as has been shown by several prospective cohort studies [7,33,59]. As nasal samples were collected at a single point of time in almost all studies included and in most cases at times convenient to the subjects during or after their work shift, no distinction between transient or persistent carriage is possible [6]. The question, whether swabs taken from HCWs outside their clinical environment might be more representative of the prevalence of persistent MRSA carriage status, cannot be answered by our data.

Routine staff screening for MRSA is highly contentious [63]. Opponents of routine staff screening argue that there is no proof of its benefit [43], that there is a risk of stigmatisation of those affected and that reliable evidence is limited on the effectiveness of staff screening for the prevention and control of MRSA in an endemic setting [13].

### Limitations

Most studies included in this review achieved only a moderate quality level. This is partly explained by our search strategy. Our review was not limited to peer-reviewed publications, as otherwise many studies would have been missed [34,39,40,47,52,56,58,61]. Due to the nature of the publications, it was not always clear whether the reporting of the study or the study itself had shortcomings. Even with this consideration, the number of good quality studies was surprisingly small. All conclusions drawn from our review should therefore be regarded with scepticism.

## Conclusions

We found that MRSA prevalence among HCWs in non-outbreak settings was no higher than carriage rates estimated for outbreaks. Our estimate is in the lower half of the range of the published MRSA rates in the endemic setting. More attention should be given to the prevention of MRSA colonisation in nursing staff, as this professional group seem to experience the highest risk for MRSA colonisation. Better standardization of screening strategies of HCWs is needed, as sampling site, frequency and timing of HCW screening are the most important reasons for misclassification.

## Competing interests

The authors declare that they have no competing interests.

## Authors’ contributions

MD developed the search strategy and conducted a title-abstract screening, independently of shared title-abstract screening by CP and AS. Full text evaluation was conducted by MD and in cases of uncertainty, discussion took place with CP and AS. MD wrote the manuscript, with significant contribution from the other authors. All authors have read and approved the final manuscript.

## Pre-publication history

The pre-publication history for this paper can be accessed here:

http://www.biomedcentral.com/1471-2334/14/363/prepub
